# Amide proton transfer-weighted imaging and T1 mapping as diagnostic tools to evaluate high-risk histopathologic phenotypes before surgery in rectal adenocarcinoma and their correlation with Ki-67 expression:a two-center study

**DOI:** 10.3389/fonc.2025.1403890

**Published:** 2025-05-19

**Authors:** Yaxin Chai, Yongchao Niu, Ruixue Cheng, Pan Liang, Jianbo Gao

**Affiliations:** ^1^ Department of Magnetic Resonance Imaging (MRI), Xinxiang Central Hospital, The Fourth Clinical College of Xinxiang Medical University, Xinxiang, China; ^2^ Department of Radiology, The First Affiliated Hospital of Zhengzhou University, Zhengzhou, China

**Keywords:** amide proton transfer (APT), T1 mapping, magnetic resonance, adenocarcinoma, Ki-67, cancer diagnosis, high-risk histopathologic phenotypes

## Abstract

**Objectives:**

To investigate the application of amide proton transfer (APT)-weighted MRI, T1 mapping in evaluating the preoperative high-risk histopathologic phenotypes of rectal adenocarcinoma and their correlation with Ki-67 expression.

**Materials and Methods:**

Retrospective collection of 178 confirmed cases of rectal adenocarcinoma from two centers (center 1: 97 cases, center 2: 81 cases). High-resolution T2WI, APT, T1 mapping, diffusion-weighted imaging (DWI), and Ki-67 staining were performed in all patients. The measured parameters included APT signal intensity (APT SI), T1 relaxation time before (native T1) and after (post-contrast T1) enhancement, and apparent diffusion coefficient (ADC). The receiver operating characteristic (ROC) curve was used to evaluate diagnostic efficiency, and Spearman correlation analysis was used to evaluate the correlation between parameters with Ki-67, respectively.

**Results:**

APT SI values were significantly different between the mucinous adenocarcinoma (MC) group and the common adenocarcinoma (AC) group in two centers (center 1: [2.64 ± 0.33%] vs. [2.22 ± 0.78%], *P*<0.05), (center 2: [3.27 ± 0.80%] vs. [2.59 ± 0.77%], *P*<0.05). In the AC group, APT SI, native T1 and ADC values were significantly different between T1–2 and T3–4 groups (center 1: [2.58 ± 0.69%] vs. [1.61 ± 0.49%], [1540 ± 150 ms] vs. [1360 ± 130 ms], [0.85 ± 0.15×10^-3^ mm^2^/s] vs. [0.99± 0.15×10^-3^ mm^2^/s], respectively, all *P*<0.05), the results were consistent with the findings of center 2. APT SI and native T1 values in the lymph node metastasis group were higher than those in the non-metastatic group (center 1: [2.49 ± 0.77%] vs. [2.07 ± 0.74%], [1540 ± 170 ms] vs. [1430 ± 160 ms], respectively, all *P*<0.05), the result were consistent with the findings of center 2. APT SI were statistically significant in evaluating lymphovascular invasion (LVI) and extramural vascular invasion (EMVI) in two centers (*P*<0.05). Ki-67 expression was correlated with APT SI (mild to medium), ADC (mild) and native T1 (mild to high) in two centers, respectively (*P*<0.05), but there was no correlation between post-contrast T1 and Ki-67 (*P*>0.05).

**Conclusion:**

APT and T1 mapping can be used to evaluate the preoperative pathological classification, TN staging, and structural invasion of rectal adenocarcinoma, which has the potential to become an imaging marker for the evaluation of high-risk histopathologic phenotypes and Ki-67 expression of rectal adenocarcinoma.

## 1 Introduction

Rectal adenocarcinoma is a common malignant tumor worldwide, accounting for approximately 9.4% of all malignant tumors, and its high morbidity and mortality seriously threaten the health and quality of life of patients (1). Neoadjuvant chemoradiotherapy (NAC) and surgical resection are the most effective treatments for rectal cancer. Direct surgical resection is usually performed for early rectal cancer, while multimodal treatment with neoadjuvant chemotherapy, radiotherapy, and total mesorectal excision (TME) is usually adopted for locally advanced rectal cancer to improve the survival rate of patients. Many factors are related to the treatment efficacy and prognosis of rectal cancer, including pathological T and N stages, differentiation degree, pathological type, and pathological grade (2, 3). Rectal mucinous adenocarcinoma (MC) has a mucin proportion greater than 50% and is a type of rectal adenocarcinoma with high malignancy and poor NAC efficacy (4). Inter- and intra-tumor heterogeneity lead to different tumor outcomes (5). European Society for Medical Oncology (ESMO) highlighted the prognostic significance of extramural vascular invasion (EMVI), lymphovascular invasion (LVI) and perineural invasion (PNI) in the rectal cancer population and included them in the evaluation system along with TNM to better predict prognosis (6). However, EMVI, LVI are usually obtained from postoperative surgical specimens, leaving only room to guide postoperative management. Therefore, preoperative knowledge of tumor and lymph node staging as well as EMVI, LVI may contribute to individualized risk stratification of resectable rectal cancer. According to the clinical practice guidelines of the ESMO (6), rectal cancer was divided into very low-risk, low-risk, medium-risk, high-risk and very high-risk stratification group according to the TN stage, depth of submucosal invasion, tumor location, mesenteric fascia (MRF), EMVI, PNI and LVI. Different risk stratifications corresponded to different treatment methods. T3-4, Lymph node metastasis (LNM), EMVI, PNI and LVI were high-risk histopathological phenotypes (7, 8), which were closely related to risk stratification. Previous studies were mostly based on one of these factors, lacking comprehensive evaluation of tumor atypia.Therefore, accurate preoperative comprehensive assessment of high-risk histopathological phenotypes of rectal cancer is more conducive to the risk stratification of rectal cancer, and more conducive to the selection of individualized treatment.

In recent years, molecular biological markers have been widely used for preoperative and prognostic prediction of rectal adenocarcinoma (9, 10). Among tumor markers, Ki-67 expression is key for the diagnosis of rectal adenocarcinoma. The occurrence and development of rectal adenocarcinoma are closely related to the apoptosis and proliferation of epithelial cells. The proliferating nuclear antigen Ki-67 is a non-histone nuclear protein that is expressed in every active phase of the cell cycle, except the quiescent phase (G0 phase) and can be used to determine the proliferative activity of tumor cells. Studies have shown that the degree of differentiation, depth of invasion, and metastasis of rectal adenocarcinoma are related to Ki-67 expression, which directly affects prognosis (11, 12). However, Ki-67 expression can only be determined using invasive biopsy or surgical pathology.

Magnetic resonance imaging (MRI) is an important imaging method for the preoperative evaluation of rectal adenocarcinoma and has been widely used for preoperative staging, curative effect evaluation, and postoperative follow-up (13, 14).Amide proton transfer (APT) is a new noninvasive molecular imaging technique based on chemical exchange saturation transfer (CEST). APT signal intensity (APT SI) is an indirect reflection of metabolic changes within the tissue determined by quantitative measurement of the magnetization transfer ratio asymmetry (MTRasym) at 3.5 ppm (15). APT SI is affected by tissue pH, T1 relaxation time, and local tissue temperature (16). APT is currently used in brain tumors for diagnosis and grading (17), efficacy evaluation (18), prognostication and survival analysis (19). Previous studies have found that the APT SI is related to the pathological grade and lymph node metastasis of rectal cancer (20–22); Akihiro et al. showed that Amide proton transfer imaging can predict the response of locally advanced rectal cancer to neoadjuvant chemotherapy (23). however, current research results vary and there is no consensus yet, with only a few studies focusing on APT and Ki-67.

Longitudinal relaxation time quantification (T1 mapping) is a quantitative MRI technique for measuring tissue T1 values. T1 relaxation time can be used to quantitatively assess the internal composition of tissues. In the past, it was commonly used to evaluate cardiomyopathy (24, 25), and now it is increasingly used to evaluate tumors. Makowski et al. suggested that T1 mapping could improve the accuracy of the Gleason score for prostate cancer (26). T1 mapping has high potential for distinguishing benign and malignant liver tumors, and native T1 is superior to diffusion weighted imaging (DWI) in distinguishing liver cysts and hemangiomas (27). Li et al. showed that native T1 could be used to distinguish between mucinous and non-mucinous adenocarcinomas of the rectum (28). Ki-67 is a protein associated with cell proliferation in growth nuclei (29). T1 is influenced by intracellular and interstitial factors. Yuan et al. showed that the native T1 value was mainly related to the number of colorectal cancer cells that proliferated (30). The native T1 value was correlated with Ki-67 expression in both meningioma and lung cancer (31, 32).

The purpose of this study was to investigate the role of APT and T1 mapping in distinguishing between low-to-high histopathologic factors (T3-4, LNM, EMVI, LVI, PNI) in rectal adenocarcinoma, and to study their correlation with Ki-67 expression, in order to provide non-invasive imaging indicators for the selection of individualized clinical treatment for rectal adenocarcinoma patients.

## 2 Materials and methods

### 2.1 Participants

**Figure 1:**
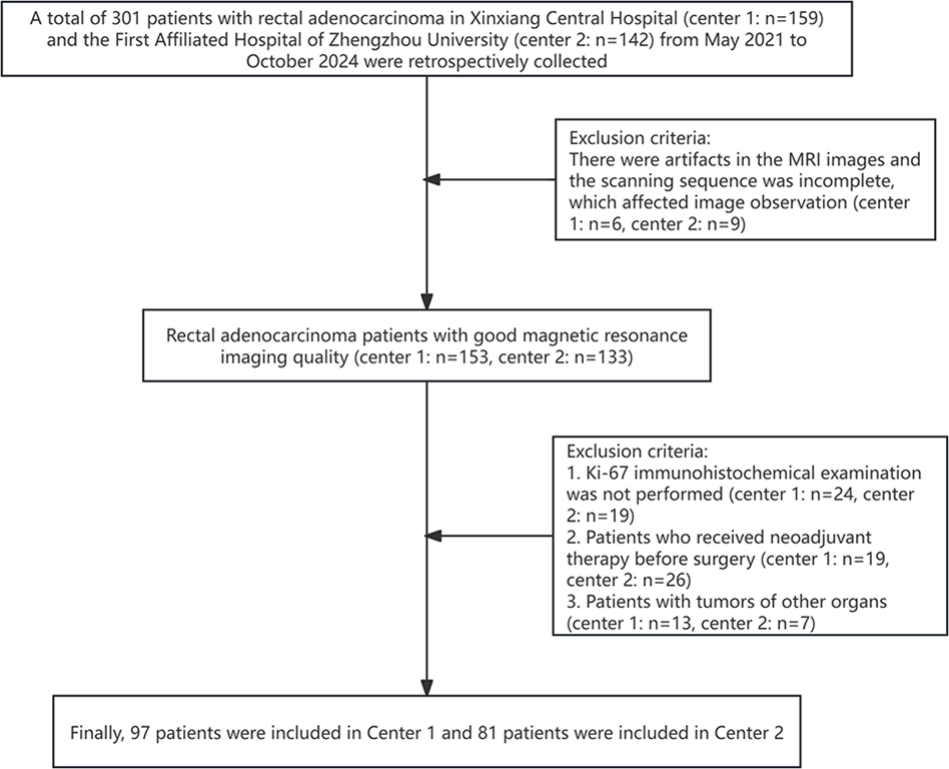
Flowchart of patient selection.

A total of 301 patients with rectal adenocarcinoma in Xinxiang Central Hospital and the First Affiliated Hospital of Zhengzhou University from May 2021 to October 2024 were retrospectively collected. The inclusion criteria were: 1. Rectal adenocarcinoma was pathologically confirmed; 2. No radiotherapy, chemotherapy, or surgery performed before the scan; 3. Complete clinical, imaging, and pathological data; 4. MRI performed within 2 weeks before surgery. The exclusion criteria were: 1. There were artifacts in the MRI images and the scanning sequence was incomplete, which affected image observation (n=15); 2. Ki-67 immunohistochemical examination was not performed (n=43); 3. Patients who received neoadjuvant therapy before surgery (n=45); 4. Patients with tumors of other organs (n=20). Finally, 97 patients were included in Center 1 and 81 patients were included in Center 2 (**Figure 1**). This study was reviewed and approved by the Ethics Committee of Xinxiang Central Hospital (2021–174) and the First Affiliated Hospital of Zhengzhou University(2024-KY-0862), all participants signed informed consent forms.

### 2.2 MRI protocol

Each patient had a liquid diet the day before the examination because a clear bowel was required 1 h before the examination. An intramuscular injection of 20 mg raceanisodamine hydrochloride (Suicheng Pharmaceutical Co, Ltd.) was given 10–15 min before the examination to suppress intestinal movement. Philips 3.0T (Ingenia Elition X, Philips Healthcare, Best, the Netherlands/Ingenia CX, Philips Healthcare, Best, the Netherlands) superconducting magnetic resonance (MR) instruments with a 32-channel phased array coil was used. Patients lied down in a supine position with an elevated head, and scanning sequences included T2WI, T1WI, DWI, APT, T1 mapping (before and after enhancement), and dynamic enhancement. The scanning parameters are listed in **Table 1**.

**Table 1:** Magnetic resonance imaging acquisition parameter.

Parameters	T2WI	DWI	APT	T1mapping(native/post-contrast)
TR/TE (ms)	3000/100	4050/48	6540/8.3	1.47/4.09
Field of view (mm^2^)	200x200	240x240	230x181	380x310
Slice thickness (mm)	5	5	5	4
No. of slices	24	24	24	24
Matrix	344x306	110x110	116x90	256x240
Spatial resolution (mm^3^)	0.6x0.7x0.4	1.8x1.8x3.0	2x2x5	1.0x1.0x4.0
b-values (s/mm^2^)	N/A	800	N/A	N/A
Bandwidth (Hz/pixel)	200	992	647.2	1530
TSE factor	15	N/A	174	N/A
Fat suppression	NO	NO	YES	NO
Acquisition time	2min45s	2min26s	4min30s	5min47s

### 2.3 Image analysis and index measurement

**Figure 2:**
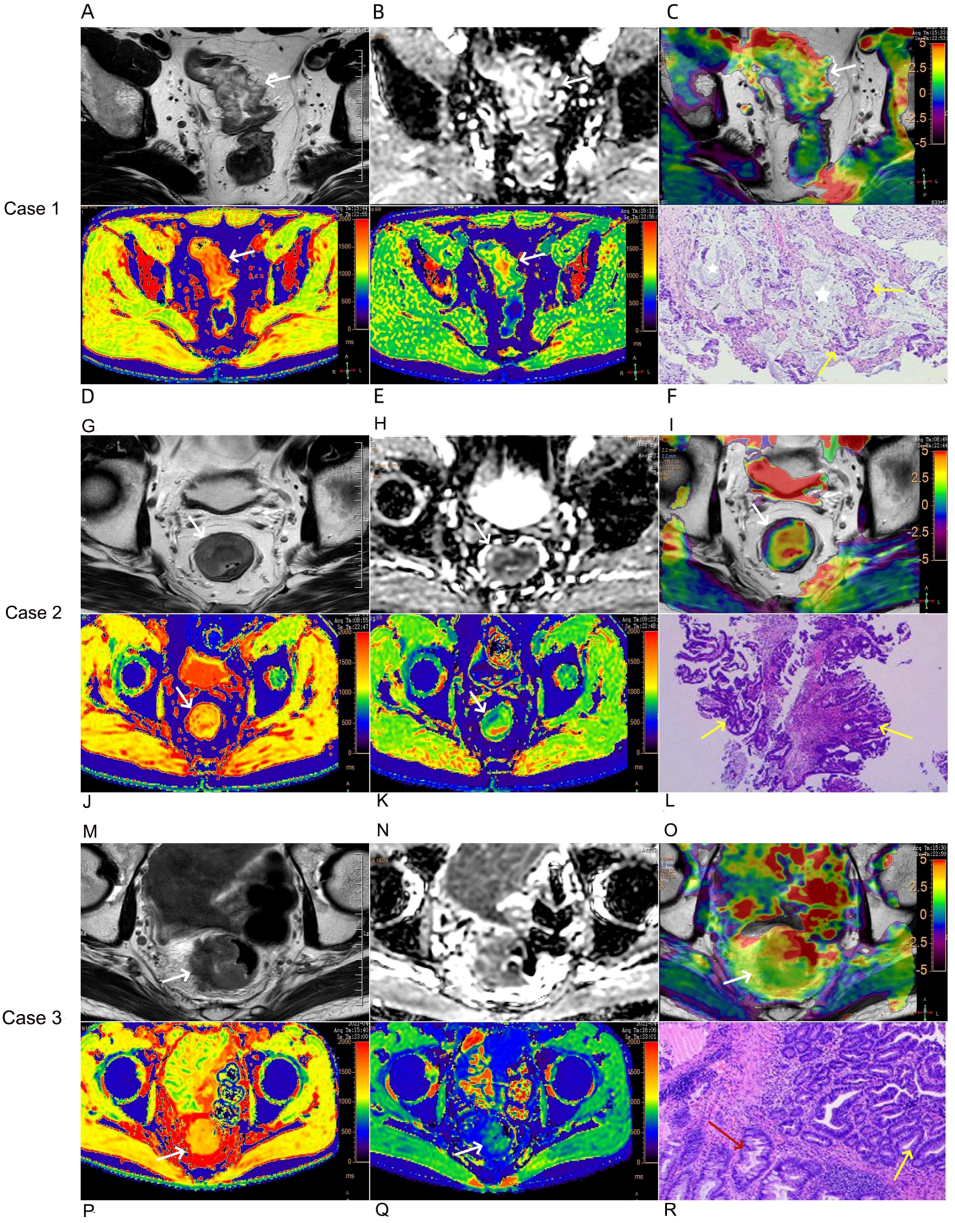
Case 1: A 76-year-old female with mucinous adenocarcinoma **(A–F)**. Oblique axial T2-weighted image showed the rectal wall thickens with a high-intensity signal **(A)**. The lesion showed a slightly high-intensity signal on the ADC map, the ADC value was 1.15×10^-3^ mm^2^/s **(B)**. APT-T2 merged image showed the mass with a mean APT SI of 3.87% **(C)**. At native T1 pseudo-color image, the lesions were mainly red, and the relaxation time of T1 was 1660 ms **(D)**. At post-contrast T1 pseudo-color image, the lesions were mainly green, and the relaxation time of T1 was 652 ms **(E)**. Pathological sections showed mucous adenocarcinoma of the rectum (HE,×200) **(F)**, tumor glandular epithelium lining around the mucinous pool, the yellow arrow represents the tumor glandular epithelium, and the white five pointed star area represents the mucinous pool. Case 2: A 52-year-old male with common adenocarcinoma,T2N0, EMVI-, PNI-, LVI- **(G–L)**. Oblique axial T2-weighted image showed the mass with a slight high-intensity signal **(G)**. The lesion showed a slightly low-intensity signal on the ADC map, the ADC value was 0.93×10^-3^ mm^2^/s **(H)**. APT-T2 merged image showed the mass with a mean APT SI of 1.17% **(I)**. At native T1 pseudo-color image, the lesions were mainly yellow, and the relaxation time of T1 was 1250 ms **(J)**. At post-contrast T1 pseudo-color image, the lesions were mainly green, and the relaxation time of T1 was 819 ms **(K)**. Pathological sections showed common adenocarcinoma of the rectum (HE,×200) **(L)**, the yellow arrow represents the tumor glandular epithelium. Case 3: A 63-year-old male with common adenocarcinoma,T3N1, EMVI+, PNI-, LVI+ **(M–R)**. Oblique axial T2-weighted image showed the mass with a slight high or iso-intensity signal **(M)**. The lesion showed a low-intensity signal on the ADC map, the ADC value was 0.87×10^-3^ mm^2^/s **(N)**. APT-T2 merged image showed the mass with a mean APT SI of 2.68% **(O)**. At native T1 pseudo-color image, the lesions were mainly yellow, and the relaxation time of T1 was 1420 ms **(P)**. At post-contrast T1 pseudo-color image, the lesions were mainly green, and the relaxation time of T1 was 717 ms **(Q)**. Pathological sections showed common adenocarcinoma of the rectum (HE,×200) **(R)**, the yellow arrow represents the tumor glandular epithelium and the red arrow represents normal epithelium.

The images were transferred to a Philips ISP v10 workstation, the APT SI, native T1, and post-contrast T1 pseudo-color images were calculated using IntelliSpace Portal post-processing software, and the APT pseudo-color images were fused with T2WI images. Each parameter was measured using a double-blind method by two radiologists who had worked for 5-8 years (YXC and YCN). The region of interest (ROI) was palced on a slice with the largest tumor diameter (the peri-rectal fat layers were excluded for ROI definitions), and combined with the T1WI, T2WI, and T1WI SPIR sequences to avoid liquefaction, bleeding, necrosis, and lipid regions. The area of interest does not include lymph nodes. In the case that lymph nodes are indistinct from the tumor, it is necessary to make a comprehensive judgment based on T2WI and T1WI images, and sketch the area of interest as close to the tumor as possible. The data in each group were measured three times and the average values were calculated. See **Figure 2**.

APT SI is the magnetization transfer ratio asymmetry at a saturation frequency offset of 3.5 ppm (MTRasym) and is calculated using the following formulae:

MTRasym(3.5,ppm)= [Ssat,(−,3.5,ppm),−,Ssat(+,3.5,ppm)]/S0  APT SI = MTRasym [Δω + 3.5 ppm](%) where S0 is the SI without the application of the saturation pulse, and Ssat is the SI after the application of the saturation pulse (33).

### 2.4 Pathological grouping

According to the pathological results, rectal adenocarcinoma was divided into rectal mucinous adenocarcinoma (MC) and common adenocarcinoma (AC). According to the staging criteria of the American Joint Committee on Cancer (AJCC) (34), early-stage cancer was defined as disease confined to the muscularis propria and late-stage cancer was defined as disease extending beyond the muscularis propria. All patients were divided into early stage (pT1–2) and late stage (pT3–4) based on the depth of postoperative pathological tumor invasion. N staging was performed according to postoperative pathology. The pN0 stage was the non-metastatic lymph node group, and the pT1–2 stage was the metastatic lymph node group. Based on pathological findings, perineural invasion (PNI), lymphovascular invasion (LVI), and extramural vascular invasion (EMVI) were divided into positive (+) and negative (–) groups.

### 2.5 Statistical analysis

SPSS 23.0 (IBM, Armonk, NY) (33) and MedCalc 15.0 (MedCalc Software, Ostend, Belgium) (35), were used for statistical analysis. Interclass correlation coefficient (ICC) was used to evaluate the consistency of measurement results of two radiologists (r≥0.75, excellent; 0.60≤r< 0.75, good; 0.40≤r< 0.60, general; r<0.40, poor). The Shapiro-Wilk test was used to evaluate whether the quantitative values were normally distributed and to determine homogeneity of variance. Measurement data conforming to a normal distribution are presented as mean ± standard deviation, while those not conforming to normal distribution were represented as median (interquartile range, IQR). Independent sample *t* test or Mann–Whitney *U* test was used to analyze differences between parameters across the groups. A receiver operating characteristic (ROC) curve was plotted to analyze the diagnostic efficiency and obtain the optimal diagnostic cutoff value. The DeLong test was used to compare differences in the area under the curve (AUC). A binary logistic model was used for the parameter fusion. Spearman correlation analysis was used to analyze the correlation between APT SI, native T1, post-contrast T1 or ADC value with Ki-67 expression (r≥0.60, high; 0.40≤r<0.60, medium; 0.20≤r<0.40, mild). *P*<0.05 was considered statistically significant.

## 3 Results

### 3.1 The clinical and pathological characteristics of the patient

Of 97 patients with rectal adenocarcinoma in center 1, there were 7 cases of rectal MC and 90 cases of AC; Among the AC group, 34 cases were T1-2 stage and 56 cases were T3-4 stage. There were 58 cases in the non-metastatic lymph node group and 32 cases in the metastatic lymph node group; 27 and 63 cases were positive and negative extramural vascular invasion (EMVI), respectively; 34 and 56 cases were positive and negative LVI, respectively; Perineural invasion (PNI) (+) group 24 cases, PNI (–) group 66 cases. Of 81 patients with rectal adenocarcinoma in center 2, there were 8 cases of MC and 73 cases of AC; Among the AC group, 24 cases were T1-2 stage and 49 cases were T3-4 stage. There were 53 cases in the non-metastatic lymph node group and 20 cases in the metastatic lymph node group; 18 and 55 cases were positive and negative extramural vascular invasion (EMVI), respectively; 21 and 52 cases were positive and negative LVI, respectively; Perineural invasion (PNI) (+) group 17 cases, PNI (–) group 56 cases (**Table 2**).

**Table 2:** Clinical and pathologic characteristics of patients studied in two centers.

Characteristics	Number of patients (Center 1)	Number of patients (Center 2)
Gender		
Male	60 (62%)	50 (62%)
Female	37 (38%)	31 (38%)
Age		
Mean age, years	59 ± 11	62 ± 12
Age range, years	32-83	26-86
Pathology		
mucinous adenocarcinoma MC	7 (7%)	8 (10%)
common adenocarcinoma AC	90 (93%)	73 (90%)
T stage (AC)		
T1-2	34 (38%)	24 (33%)
T3-4	56 (62%)	49 (67%)
N stage (AC)		
N0	58 (64%)	53 (73%)
N1-2	32 (36%)	20 (27%)
Extramural vascular invasion, EMVI (AC)		
Positive	27 (30%)	18 (25%)
Negative	63 (70%)	55 (75%)
Lymphovascular invasion, LVI (AC)		
Positive	34 (38%)	21 (29%)
Negative	56 (62%)	52 (71%)
Perineural invasion, PNI (AC)		
Positive	24 (27%)	17 (23%)
Negative	66 (73%)	56 (77%)
Ki-67 (AC, MC)	97 (100%)	81 (100%)

### 3.2 Consistency test of APT SI, native T1, post-contrast T1 and ADC values measured by two physicians

The ICC values of the APT SI, native T1, post-contrast T1, and ADC measured by the two physicians from center 1 were 0.86 (95%CI: 0.80~0.91), 0.84 (95%CI: 0.77~0.89), 0.79 (95%CI: 0.69~0.85), and 0.84 (95%CI: 0.76~0.89), respectively. The ICC values from center 2 were 0.84 (95%CI: 0.76~0.90), 0.85 (95%CI: 0.77~0.90), 0.97 (95%CI: 0.95~0.98), and 0.92 (95%CI: 0.88~0.95), respectively, indicating excellent consistency. The average value of the two measurements was used as the evaluation index.

### 3.3 Comparison and diagnostic efficiency of APT SI, native T1, post-contrast T1, and ADC parameters between MC group and AC group

**Figure 3:**
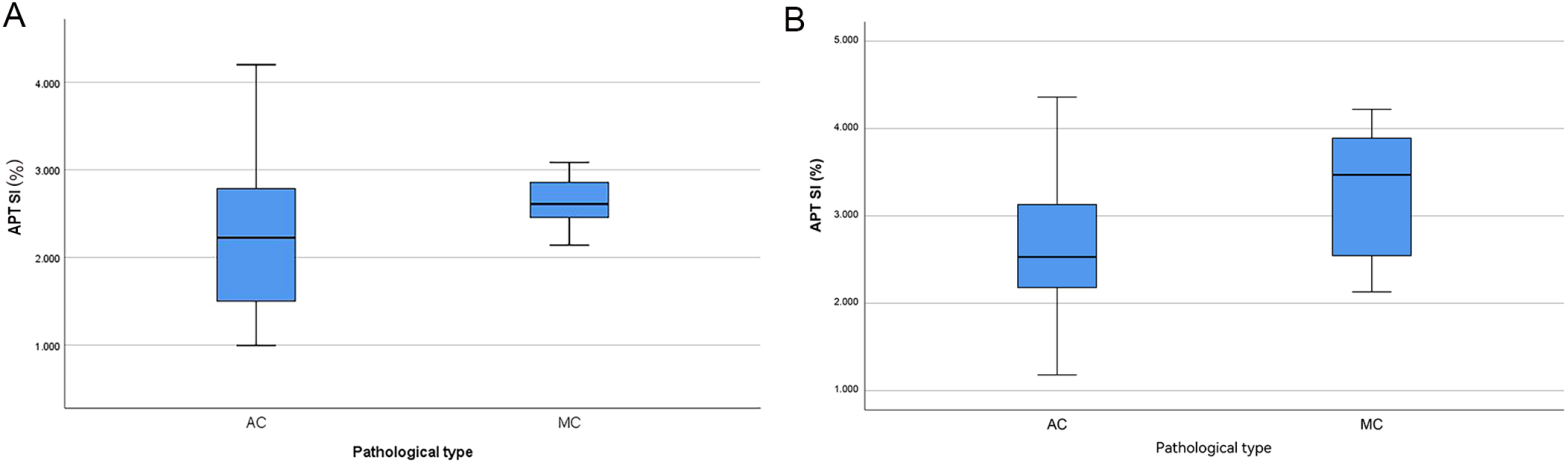
Boxplots of APT SI value in different pathological type (**A**: center 1, **B**: center 2), the APT SI value was significantly higher in MC than in AC.

**Figure 4:**
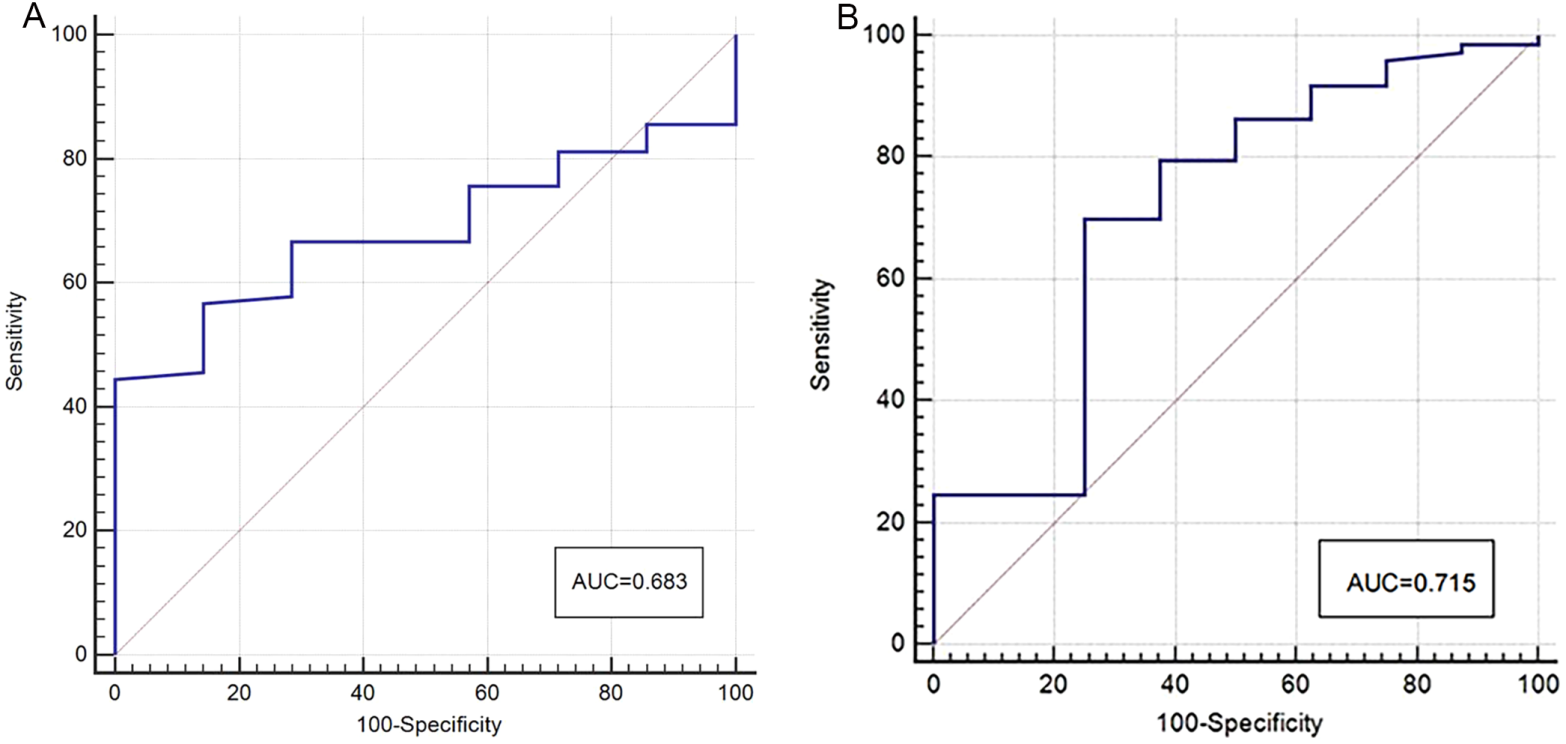
ROC curves of APT SI for discrimination between MC and AC (**A**: center 1, **B**: center 2), the AUC was 0.68 (center 1) and 0.72 (center 2) respectively.

The APT SI values of the two centers showed a statistically significant difference between the two groups, with the APT SI value in the MC group being larger than that in the AC group. However, only in Center 2, a statistically significant difference was found for native T1 values between the two groups, as shown in **Table 3** and **Figure 3**. The AUC of APT SI for diagnosing rectal cancer types in center 1 is 0.68, and the AUC for center 2 is 0.72. (**Figure 4**). All other parameters (past-contrast T1, ADC) did not show statiscally significant differences between MC and AC.

**Table 3:** Comparison of APT, T1 mapping and ADC parameters between MC and AC groups in two centers ( $\bar{x}$ ± s).

Groups	APT SI (%)	Native T1 (ms)	Post-contrast T1 (ms)	ADC (x10^-3^mm^2^/s)
Center 1				
MC (7)	2.64 ± 0.33	1550 ± 220	709 ± 110	0.99 ± 0.22
AC (90)	2.22 ± 0.78	1470 ± 170	734 ± 150	0.90 ± 0.16
*t*	-2.812	-1.178	0.414	-1.469
*P*	0.016	0.242	0.680	0.145
Center 2				
MC (8)	3.27 ± 0.80	1660 ± 190	464 ± 65	0.77 ± 0.14
AC (73)	2.60 ± 0.77	1390 ± 240	524 ± 130	0.91 ± 0.28
*t*	-2.340	-3.122	1.334	1.369
*P*	0.022	0.003	0.185	0.175

### 3.4 Comparison of values of APT SI, native T1, post-contrast T1, and ADC between different sub-groups in the AC groups

In center 1: APT SI and native T1 were significantly different in the T, N, LVI, and EMVI groups (*P*<0.05); ADC value in T3–4 group was lower than that in T1–2 group, while that in EMVI+ group was lower than in EMVI-, the difference was statistically significant (*P*<0.05); APT SI, native T1, post-contrast T1, and ADC did not show statistical significance in evaluating the PNI (*P*>0.05). (**Table 4**).

**Table 4:** The comparison of APT SI, native T1, post-contrast T1, ADC values in different groups of AC in center 1.

Groups	APT SI (%)	Native T1 (ms)	Post-contrast T1 (ms)	ADC (x10^-3^mm^2^/s)
T stage				
T1-2 (34)	1.61 ± 0.49	1360 ± 130	771 ± 150	0.99 ± 0.15
T3-4 (56)	2.58 ± 0.69	1540 ± 150	710 ± 160	0.85 ± 0.15
*t*	-7.178	-5.708	1.824	4.532
*P*	0.000	0.000	0.072	0.000
N stage				
N0 (58)	2.07 ± 0.74	1430 ± 160	729 ± 150	0.91 ± 0.13
N1-2 (32)	2.49 ± 0.77	1540 ± 170	741 ± 160	0.88 ± 0.21
*t*	-2.533	-2.961	-0.345	0.849
*P*	0.013	0.004	0.731	0.400
Perineural invasion (PNI)				
Positive (24)	2.33 ± 0.85	1500 ± 190	737 ± 160	0.87 ± 0.19
Negative (66)	2.18 ± 0.75	1460 ± 160	732 ± 150	0.91 ± 0.16
*t*	-0.825	-0.979	-0.135	1.022
*P*	0.411	0.330	0.893	0.310
Lymphovascular invasion (LVI)				
Positive(34)	2.49 ± 0.83	1530 ± 180	739 ± 150	0.88 ± 0.18
Negative(56)	2.05 ± 0.70	1430 ± 150	730 ± 160	0.91 ± 0.16
*t*	-2.697	-2.854	-0.261	0.853
*P*	0.008	0.005	0.795	0.396
extramural vascular invasion(EMVI)				
Positive(27)	2.56 ± 0.74	1540 ± 160	704 ± 180	0.85 ± 0.18
Negative(63)	2.07 ± 0.75	1440 ± 160	746 ± 140	0.93 ± 0.15
*t*	-2.858	-2.764	1.053	2.194
*P*	0.005	0.007	0.299	0.031

In center 2: APT SI and native T1 were significantly different in the T, N, and LVI groups (*P*<0.05); ADC value in T3–4 group was lower than that in T1–2 group (*P*<0.05); APT SI and ADC were statistically significant in evaluating EMVI (*P*<0.05); APT SI, native T1, post-contrast T1, and ADC did not show statistical significance in evaluating the PNI (*P*>0.05). (**Table 5**).

**Table 5:** The comparison of APT SI, native T1, post-contrast T1, ADC values in different groups of AC in center 2.

Groups	APT SI (%)	Native T1 (ms)	Post-contrast T1 (ms)	ADC (x10^-3^mm^2^/s)
T stage				
T1-2 (24)	2.31 ± 0.64	1080 ± 65	512 ± 150	1.09 ± 0.29
T3-4 (49)	2.74 ± 0.80	1490 ± 210	530 ± 110	0.85 ± 0.25
*t*	-2.289	-12.499	0.572	2.747
*P*	0.025	0.000	0.569	0.008
N stage				
N0 (53)	2.27 ± 0.58	1290 ± 210	525 ± 130	0.97 ± 0.27
N1-2(20)	3.48 ± 0.47	1520 ± 310	522 ± 100	0.75 ± 0.22
*t*	-8.394	-3.016	-0.099	3.292
*P*	0.000	0.006	0.922	0.072
Perineural invasion (PNI)				
Positive (17)	2.52 ± 0.79	1450 ± 180	495 ± 110	0.94 ± 0.35
Negative (56)	2.62 ± 0.77	1330 ± 270	533 ± 130	0.90 ± 0.26
*t*	-0.463	-1.728	1.112	-0.524
*P*	0.645	0.088	0.270	0.602
Lymphovascular invasion (LVI)				
Positive (21)	3.06 ± 0.61	1530 ± 240	551 ± 130	0.78 ± 0.23
Negative (52)	2.41 ± 0.75	1280 ± 230	513 ± 120	0.96 ± 0.28
*t*	-3.508	-4.099	-1.155	2.563
*P*	0.001	0.000	0.252	0.012
extramural vascular invasion (EMVI)				
Positive (18)	3.10 ± 0.76	1430 ± 310	532 ± 100	0.80 ± 0.25
Negative (55)	2.43 ± 0.70	1330 ± 230	520 ± 133	0.95 ± 0.28
*t*	-3.408	-1.289	-0.290	2.053
*P*	0.001	0.210	0.773	0.044

### 3.5 Diagnostic efficiency of APT SI, native T1, post-contrast T1 and ADC values among different sub-groups within the AC group

**Figure 5:**
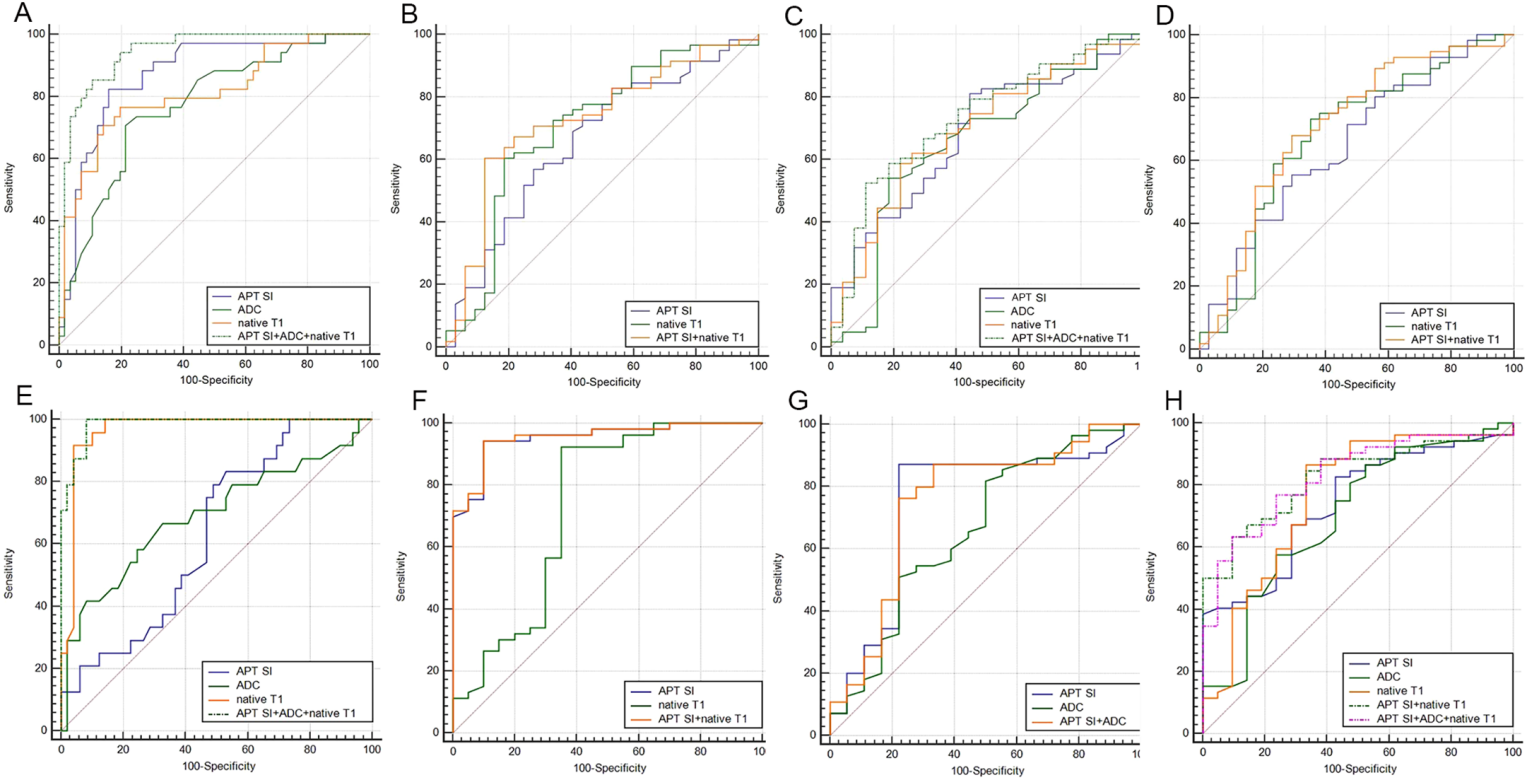
ROC curves of APT SI, ADC, native T1 and APT SI+ADC+native T1 for discrimination between T1-2 and T3-4 (center1: **A** center 2: **E**, APT SI+ADC+ native T1 had the highest AUC in two centers(center 1: 0.95, center 2: 0.99); ROC curves of APT SI, native T1 and APT SI+native T1 for discrimination between N0 and N1-2 (center1: **B** center 2: **F**), in the two centers, the AUC of APT SI+native T1 was higher than that of APT SI and native T1, 0.73 (center 1) and 0.96 (center 2), respectively; ROC curves of APT SI, ADC, native T1, APT SI+ADC and APT SI+ADC+native T1 for discrimination between EMVI+ and EMVI- (center1: **C**; center 2: **G**), in the EMVI evaluation of Center 1, APT SI, native T1 and ADC all had statistical significance, and the combined AUC of the three was the highest (0.73). In the evaluation of center 2, only APT SI and ADC had statistical significance, and APT SI+ADC had the highest AUC (0.75), which was higher than the combined AUC of center 1; ROC curves of APT SI, native T1, ADC, APT SI+native T1 and APT SI+ADC+native T1 for discrimination between LNI+ and LNI- (center1: **D**; center 2: **H**), in the LVI evaluation of Center 1, APT SI and native T1 had statistical significance, and the combined AUC of the two was the highest (0.71); In the evaluation of center 2, APT SI, native T1 and ADC all had statistical significance, and APT SI+ADC had the AUC of 0.82, which was higher than the combined AUC of center 1. Additionally, the highest AUC for APT S1+native T1+ADC is 0.83.

Comparing group T1–2 with T3–4 group: In center 1, the joint probability *P*-value of APT SI, ADC, and native T1 had the highest AUC (0.95), with diagnostic sensitivity and specificity of 89% and 85%, respectively (**Figure 5A**); In center 2, the joint probability *P*-value of APT SI, ADC, and native T1 had the highest AUC (0.99), with diagnostic sensitivity and specificity of 100% and 92%, respectively (**Figure 5E**);. Comparing group N0 with group N1–2: In center 1, the joint probability *P-*value AUC of APT SI and native T1 was the highest (0.73) (**Figure 5B**); In center 2, the joint probability *P-*value AUC of APT SI and native T1 was the highest (0.96) (**Figure 5F**). Comparing group EMVI+ with group EMVI-: In center 1, the AUC of the joint probability *P*-value of APT SI, ADC, and native T1 was higher than that of each parameter alone, but its diagnostic specificity was lower than that of APT SI (**Figure 5C**); In center 2, the AUC of the joint probability *P*-value of APT SI and ADC was higher than that of each parameter alone, but its diagnostic sensitivity and specificity were lower than that of APT SI (**Figure 5G**). Comparing group LVI+ with group LVI-: In center 1, the joint probability *P*-value of APT SI and native T1 had the highest AUC, but its diagnostic specificity was lower than that of native T1(**Figure 5D**); In center 2, the joint probability *P*-value of APT SI, ADC and native T1 had the highest AUC, the specificity was also the highest, and the sensitivity was lower than that of the three alone (**Figure 5H**). The diagnostic efficiency of each parameter and the optimal diagnostic threshold are listed in **Tables 6**, **7**.

**Table 6:** Comparison of diagnostic efficiency of APT SI, native T1, post-contrast T1 and ADC for AC groups in center 1.

Category	*P* value	AUC (95% CI)	Threshold	Sensitivity (%)	Specificity (%)
T1-2 *vs* T3-4					
APT SI	0.000	0.87 (0.78 - 0.93)	1.93	82	84
ADC	0.000	0.77 (0.67 - 0.85)	0.92	79	71
native T1	0.000	0.81 (0.72 - 0.89)	1430	80	76
APT SI+ADC+native T1	0.000	0.95 (0.88-0.98)	Not applicable	89	85
N0 *vs* N1-2					
APT SI	0.008	0.66 (0.55-0.76)	2.71	47	83
native T1	0.000	0.71 (0.60-0.78)	1440	81	60
APT SI+native T1	0.000	0.73 (0.62-0.82)	Not applicable	88	60
EMVI(+) *vs* (-)					
APT SI	0.002	0.68 (0.58 - 0.78)	2.69	56	81
ADC	0.017	0.66 (0.55 - 0.76)	0.91	81	54
native T1	0.001	0.69 (0.59 - 0.79)	1450	78	59
APT SI+ADC+native T1	0.000	0.73 (0.63 - 0.82)	Not applicable	89	52
Lymphovascular invasion (+) *vs* (-)					
APT SI	0.012	0.65 (0.54-0.75)	2.14	71	55
native T1	0.003	0.68 (0.57-0.78)	1500	65	73
APT SI+native T1	0.000	0.71(0.61-0.80)	Not applicable	71	68

**Table 7:** Comparison of diagnostic efficiency of APT SI, native T1, post-contrast T1 and ADC for AC groups in center 2.

Category	*P* value	AUC (95% CI)	Threshold	Sensitivity (%)	Specificity (%)
T1-2 *vs* T3-4					
APT SI	0.044	0.63 (0.51 - 0.74)	2.67	83	47
ADC	0.008	0.69 (0.57 - 0.80)	0.89	67	67
native T1	0.000	0.96 (0.89 - 0.99)	1170	92	96
APT SI+ADC+native T1	0.000	0.99 (0.92-0.10)	Not applicable	100.	92
N0 *vs* N1-2					
APT SI	0.000	0.95 (0.88-0.99)	2.99	94	90
native T1	0.003	0.73 (0.62-0.83)	1580	93	65
APT SI+native T1	0.000	0.96 (0.88-0.99)	Not applicable	94	90
EMVI(+) *vs* (-)					
APT SI	0.001	0.75 (0.63 - 0.84)	2.74	87	78
ADC	0.043	0.66 (0.54 - 0.77)	0.71	82	50
APT SI+ADC	0.039	0.75 (0.63 - 0.84)	Not applicable	73	50
Lymphovascular invasion (+) *vs* (-)					
APT SI	0.000	0.74 (0.63-0.84)	2.99	83	57
ADC	0.005	0.70 (0.58-0.80)	0.70	87	48
native T1	0.000	0.76 (0.65-0.86)	1490	87	67
APT SI+native T1	0.000	0.82 (0.72-0.90)	Not applicable	63	90
APT SI+ADC+native T1	0.000	0.83 (0.72-0.91)	Not applicable	73	90

### 3.6 Correlation between APT SI, native T1, post-contrast T1, and ADC values and Ki-67 expression in rectal cancer

**Figure 6:**
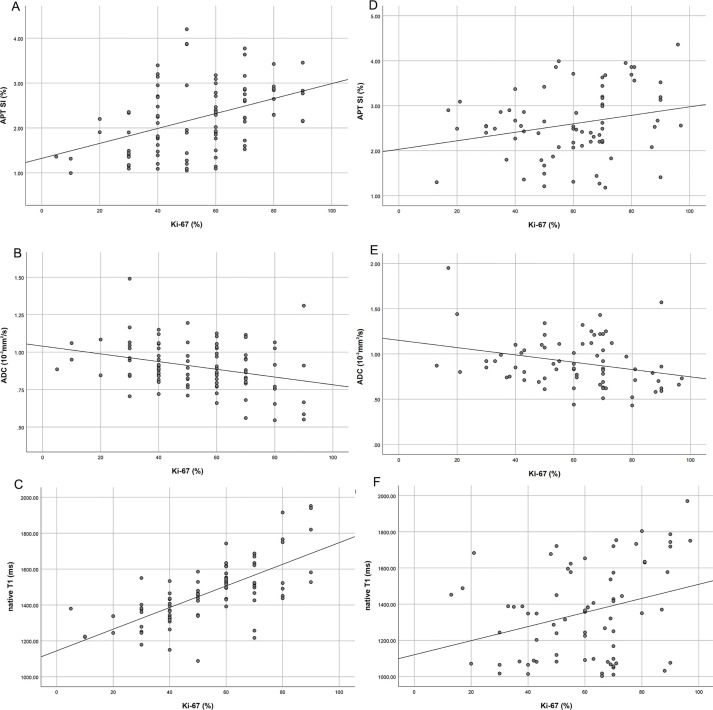
Scatter plot of correlation analysis between Ki-67 and APT SI (center1: **A**; center 2: **D**), APT SI was positively correlated with KI-67 expression, but the correlation coefficient of center 2 (0.242) was lower than that of center 1 (0.416); Scatter plot of correlation analysis between Ki-67 and ADC (center1: **B**; center 2: **E**), ADC was negatively correlated with Ki-67 expression, but the correlation coefficient of center 2 was slightly higher than that of center 1; Scatter plot of correlation analysis between Ki-67 and native T1 (center1: **C**; center 2: **F**), native T1 was positively correlated with Ki-67 expression, but the correlation coefficient of center 2 (0.300) was significantly lower than that of center 1 (0.712).

Center 1: Ki-67 expression 70.00 (60.00~80.00)%; Center 2: Ki-67 expression 70.00 (47.50~80.00)%. APT SI mild to medium correlation with Ki-67 expression (center 1: r=0.416, *P*<0.05; center 2: r=0.242, *P*<0.05) (**Figures 6A, D**), and ADC mild correlation with Ki-67 expression (center 1: r=-0.233, *P*<0.05; center 2: r=0.-289, *P*<0.05) (**Figures 6B, E**). Native T1 mild to high correlation with Ki-67 expression (center 1: r=0.712, *P*<0.05; center 2: r=0.300, *P*<0.05) (**Figures 6C, F**), whereas no correlation was observed between post-contrast T1 and Ki-67 expression (center 1: r=-0.060, *P*>0.05; center 2: r=-0.138, *P*>0.05). Linear regression analysis showed that APT SI and native T1 were independent factors influencing Ki-67 expression (center 1). Linear regression analysis showed that APT SI was independent factor influencing Ki-67 expression (center 2).

## 4 Discussion

Rectal adenocarcinoma (RA) is a common malignancy of the digestive tract. Although the prevention, screening, and treatment of rectal cancer have significantly improved, it still threatens the health of people worldwide. The prognosis of rectal cancer depends on the size, type, stage, lymph node status and peripheral invasion of the tumor, among which T3-4, TNM, EMVI, LVI and PNI are high-risk histopathological phenotypes of rectal adenocarcinoma, which directly affect the risk stratification of rectal adenocarcinoma. We combined APT and T1 mapping of two centers to conduct a comprehensive study on the high-risk histopathologic features of rectal adenocarcinoma, and the results showed that the APT SI can be used to distinguish between MC and AC. Additionally, in the AC group, findings revealed that APT SI and native T1 values can be used to evaluate the T stage, N stage, LVI. ADC may also be used to evaluate the preoperative T stage and EMVI of rectal adenocarcinoma. The combination of APT and T1 mapping can improve diagnostic efficiency. However, APT and T1 mapping have limitations in assessing PNI for rectal adenocarcinoma.

Our research indicates that the APT SI values of the MC group in both centers were higher than that of the AC group. The APT SI value was determined by endogenous free proteins and peptides and was also influenced by the pH value of the internal environment (36). MC have special histological manifestations; there is an obvious mucous lake inside the tumor, which is a malignant tumor with hypersecretion of mucus. We hypothesize that the mucous lakes, accompanied by free proteins and a large amount of mucous secretion, as well as changes in the pH value of the internal environment, resulting in an increase in the APT SI value. This is consistent with the research results of Li et al. (36), and the APT values of center 2 groups are close to them. However, in this study, there was a large difference in APT values between the two centers. The APT value of the MC group in center 1 is close to that of the AC group in center 2. Yang et al. showed that in non-mucous rectal adenocarcinoma, the APT value of tumors in the Kirsten rat sarcoma (KRAS) -mutant group was higher than that in the wild type group (3.32% ± 0.69% vs. 2.23% ± 0.83%, *P*<0.001), and the cutoff value was 2.4% (37). KRAS mutations can activate multiple intracellular pathways, promoting cell growth, proliferation, differentiation, survival, and cytokine secretion (38), all the above mentioned processes induce the increase of cellular protein content in KRAS-mutant rectal tumors, resulting in APTw hyperintensity. We hypothesize that factors such as unanalyzed KRAS mutation, parameter differences between the two central machine models, possible fat interference within adenocarcinoma (MC and AC), and selection bias may lead to the variability of APT values. In center 2, native T1 also showed statistical significance in distinguishing rectal AC from MC, which is inconsistent with the results of the center 1. Tissue T1 relaxation time is associated with a variety of biological factors, such as macromolecule concentration, water binding status, and tissue water content. The characteristic of MC is that excessive secretion of mucous by tumor cells leads to prolonged relaxation time of T1, while AC is often accompanied by more epithelial cells and proliferating cells in the tumor, which also leads to prolonged relaxation time of T1, which could result in instability of native T1 in evaluating AC and MC.

In the AC group, the APT SI values of both centers were statistically significant when evaluating the T and N groups, which is consistent with the results of Chen et al. (21), who showed that APT SI had statistically significant differences during the T2, T3, and T4 stages of rectal cancer and that the APT SI value gradually increased with the progression in stage (20), indicating that the increased aggressiveness of the tumor is often accompanied by the active proliferation of tumor cells, increase in tumor blood vessels, and increase in intracellular free proteins, ultimately increasing the APT SI value. According to a study by Li et al., the APT SI of the highly differentiated group was significantly higher than that of the poorly differentiated group (36), which is inconsistent with the results of this study. We hypothesize that this might have been caused by the different sample selections and internal composition ratios of tumors in the two studies. In this study, the native T1 value of stage T3–4 was higher than that of the stage T1–2 group, and the native T1 value of group N1–2 was higher than that of group N0. Li et al. showed that patients with advanced cervical squamous cell carcinoma had higher native T1 values (39). Native T1-based radiomics can be used for the preoperative evaluation of prostate cancer (26). T1 relaxation time has high diagnostic sensitivity in distinguishing benign from malignant breast lesions (40). T1 mapping can also be used to identify head and neck squamous cell carcinoma (HNSCC) patients with lymph node metastasis (41). T1 mapping is also an endogenous marker of tissues and is related to many biological factors, such as macromolecule concentration, water-binding state, and tissue water content (42). At the pathological level, T1 positively correlated with increased tumor water content and cell proliferation but negatively correlated with tumor necrosis (43). In this study, the advanced T stages and lymph node metastasis groups showed higher native T1 values, which may be due to the active proliferation of tumor cells, abundant extracellular matrix, and increased levels of macromolecular substances. In other clinical studies, elevated T1 values have been associated with high-risk histopathological features and poor prognoses (44). At the two centers, ADC can also distinguish T1-2 and T3-4, with AUCs of 0.77 and 0.69, respectively. The combined probability *P*-value AUC of APT SI, ADC and native T1 was the highest, and the diagnostic sensitivity and specificity were higher than those of the three alone. In terms of preoperative N staging evaluation, the diagnostic efficiency of APT SI combined with native T1 was the highest, while ADC cannot be used for evaluation. This indicates that both APT SI and native T1 could be used for preoperative T and N staging evaluation of rectal cancer, and that the combination of the two could improve diagnostic efficiency.

In two centers, the APT SI and native T1 values in LVI+ groups were higher than those in LVI- groups, respecticely. But in the center 2, the ADC also could distinguish between LVI+ and LVI-, this is inconsistent with the findings of Center 1. Yuan et al. (30), suggesting that the ADC is unstable in evaluating LVI. In two centers, the APT SI of EMVI+ group was higher than that of EMVI- group, and the ADC value was lower than that of EMVI- group; But in center 1, native T1 can distinguish between EMVI+ and EMVI-, which is inconsistent with the results reported by Li et al. (28, 45). First, we hypothesize that there are biases in patient enrollments in the two studies; Second, cancer tissues are often accompanied by cell necrosis, and large moles of molecular substances are released into the space around cells, which further shrinks the extracellular space; Third, the decrease in water content in the tumor was accompanied by a change in the free protein content; These factors affected native T1 and APT SI values. In patients with cervical cancer, the extracellular volume (ECV) based on T1 values can be used to assess LVI (46). A histogram based on the APT SI showed that LVI of rectal adenocarcinoma was associated with an increased APT SI value (47). LVI and EMVI are important prognostic factors in rectal cancer and are directly related to the choice of treatment, postoperative recurrence, and metastasis. The results of this study indicate that APT SI and native T1 can be used to evaluate LVI, and the combination of the two can improve the diagnostic efficiency. APT SI and ADC are stable in evaluating EMVI, and the combination of the two can improve diagnostic efficiency.In two centers, neither APT nor T1 mapping was statistically significant in assessing PNI for rectal adenocarcinoma, this is consistent with the findings of Li et al. (28), which may be because the tumor microenvironment reflected by the APT or T1 mapping parameters is not sufficient to cause significant changes in perineuronal invasion. In addition, T1 values are underestimated because they are susceptible to slight systematic bias, and unpredictable tissue movements can also affect T1 values, resulting in T1 value variability (48).

In this study, both APT SI and native T1 values positively correlated with Ki-67 expression, whereas ADC negatively correlated with Ki-67 expression, which is consistent with previous studies (20, 30). Ki-67 is an important prognostic factor in patients with rectal cancer and can reflect tumor aggressiveness. High expression of Ki-67 is often caused by an increase in cell number, enlargement of the nucleus, increase in macromolecular proteins, and increase in tumor vessels (49). The increase in macromolecular proteins and hemoglobin lead to an increase in the APT SI value; therefore, the APT SI value positively correlated with Ki-67 expression. The proliferation of tumor cells, an increase in the nucleoplasma ratio, a decrease in extracellular space and water content led to an increase in the native T1 value (50). The more active the proliferation of malignant tumor cells are, the greater the degree of limited diffusion, and the lower the ADC value. ADC negatively correlated with Ki-67 expression, which is consistent with the results of El Sayed et al. (51). Linear regression analysis showed that APT SI and native T1 in center 1 were independent factors affecting Ki-67 expression, while APT SI in center 2 was an independent factor affecting Ki-67 expression. In addition, there was a significant difference in the correlation coefficient between native T1 and Ki-67 in the two centers. In summary, APT SI can more stably evaluate Ki-67 expression.

Post-contrast T1 mapping refers to the longitudinal relaxation time image after injection of contrast agent. It can be used to distinguish between radionecrosis and tumor recurrence, and when contrast agents accumulate in tumor tissue outside the blood vessels, it causes the tissue relaxation time T1 to shorten (52). In this study, post-contrast T1 could not be used to evaluate TN stage and peripheral invasion of rectal adenocarcinoma, and there was no correlation with Ki-67 expression,which was inconsistent with the results of previous studies. Yuan et al. ‘s study showed that post-contrast T1 value could evaluate venous invasion and nerve invasion of rectal cancer, and there is a significant positive correlation between native T1 and Ki-67 (30). Liu et al. showed that the post-contrast T1 can distinguish between rectal MC and AC (53). The reason for this may be that the continuous proliferation and division of tumor cells will lead to a decrease in extracellular volume, and the dense structure of the tumor will cause pharmacokinetic changes, all of which will result in a prolonged T1 relaxation time. A large number of tumor neovascularization are immature and damaged, and contrast agents cannot effectively and quickly clear them, which can shorten T1 relaxation time. Meanwhile, the extracellular space of rectal cancer may contain complex components such as cystic degeneration and necrosis, which can also cause changes in T1 relaxation time. Therefore, this may leads to differences in the evaluation of post-contrast T1 values for rectal cancer classification, EMVI, LVI, KI-67 expression.

This study had some limitations. Frist, the sample size is small, and more cases should be used to confirm the validity of our results in the future. Second, when measuring quantitative parameters in this study, the ROC was placed on a slice with the largest tumor diameter, which was simple and practical to operate but could not represent the overall level of the tumor. Third, owing to the retrospective nature of the study, selection bias was inevitable, hence, findings from the study need to be further verified by prospective studies. Fourth, Mtrasym-based APTw imaging is highly sensitive to fat content, although we avoided placing ROI on the fat-affected region of the tumor by combining multiple sequences, interference with APT values by fat was still inevitable. Finally, In this study, APT SI can distinguish between MC and AC groups, but the selection of cutoff value is quite different. In future studies, reference tissue in the abdomen can be selected to control possible bias in APT SI values.

This study suggests that the APT SI can be used to distinguish between rectal MC and AC before surgery, and both APT SI and native T1 can be used to assess the T stage, N stage, and LVI of rectal adenocarcinoma. The combination of these two methods can improve the efficiency of diagnosis. APT SI and ADC can be used to assess EMVI status, while the assessment ability of native T1 is controversial. In addition, APT SI can noninvasively predict Ki-67 expression before surgery. In conclusion, APT and T1 mapping can be used to evaluate high-risk histopathologic phenotypes of rectal adenocarcinoma other than PNI, providing physicians with non-invasive and accurate preoperative tools to support clinical decision making.

## Data Availability

The datasets presented in this study can be found in online repositories. The names of the repository/repositories and accession number(s) can be found in the article/supplementary material.
